# Oxytocin effects on socially transmitted food preferences are moderated by familiarity between rats

**DOI:** 10.1007/s00213-024-06682-x

**Published:** 2024-09-25

**Authors:** Irina Noguer-Calabús, Sandra Schäble, José Dören, Tobias Kalenscher

**Affiliations:** Comparative Psychology, Institute of Experimental Psychology, Heinrich-Heine University Düsseldorf, Universitätsstraße 1, 40225 Düsseldorf, Germany

**Keywords:** Familiarity, Food preference, Group bias, Oxytocin, Reward revaluation, Social behavior.

## Abstract

**Rationale:**

In the socially transmitted food preference (STFP) paradigm, rats change their preference for food rewards after socially interacting with a conspecific who has been fed with the originally non-preferred food. Here, we asked if oxytocin (OXT), a neuropeptide known for its role in social affiliation and social behavior, plays a role in STFP. Since OXT’s influences on social behavior can be familiarity-dependent, we further asked if OXT effects on STFP are moderated by the familiarity between rats.

**Objectives:**

Does OXT modulate rats’ socially transmitted food choices in a familiarity-dependent way.

**Methods:**

We systemically injected either vehicle, low-dose (0.25 mg/kg) of OXT, or large-dose (1.0 mg/kg) of OXT before social interaction with either a familiar cagemate (in-group) or an unfamiliar conspecific from a different cage (out-group).

**Results:**

We found an intergroup bias in STFP: vehicle-treated rats showed larger socially transmitted changes in food preference in the out-group than the in-group condition. OXT modulated STFP in a familiarity-dependent way: OXT prevented the increase in the consumption of the non-preferred food in the out-group, and decreased the consumption of the preferred food in the in-group. These effects were dose-dependent and observed under acute OXT action, but also on the subsequent day when acute OXT effects dissipated, suggesting long-lasting social learning effects of OXT. Additional analyses suggest that the familiarity and dose-dependent effects of OXT on STFP cannot be attributed to OXT’s anorexic actions or differences in the duration of the social interactions.

**Conclusions:**

OXT modulates STFP in a familiarity-dependent way.

**Supplementary Information:**

The online version contains supplementary material available at 10.1007/s00213-024-06682-x.

## Introduction

What we eat is a daily decision that is influenced by our knowledge of the available resources and our dietary preferences. To make these decisions, we gather relevant information either from our own experience or through social learning. Relying on social information to choose food has proven to be an adaptive foraging strategy in many situations and in several species (Kendal et al. 2005). To operationalize social food learning in animals in a laboratory setup, Galef and Wigmore (1983) established the socially transmitted food preference (STFP) paradigm where one rat (the observer) reveals a preference for a flavored food after interacting with a demonstrator who recently ate it. Years of research using the STFP paradigm have provided solid evidence for socially transmitted food preferences, which occur independent of the observer’s energy state (food-deprived or fed ad-libitum) or the demonstrator’s characteristics, such as health (poisoned, anesthetized or controls) or age (Galef et al. 1983, 1984; Galef and Wigmore 1983; Galef and Whiskin 2004, 2008a). Aligning food preferences to those of conspecifics is a phenomenon found in many mammals including humans (Nook and Zaki 2015).

Here, we asked what the psychopharmacological mechanism of socially transmitted food preference is. One strong neuromodulator candidate is oxytocin (OXT). OXT is a neuropeptide primarily synthesized in the paraventricular hypothalamic nucleus and the supraoptic nucleus of the hypothalamus that modulates neural activity in many parts of the brain (Ferris et al. 2015; Salvi et al. 2018; Liu et al. 2021). It is prominently involved in social behavior, such as reproduction, social recognition and memory, pair bonding, and prosociality, as well as the regulation of fear, anxiety and food consumption (Jurek and Neumann 2018; Sakamoto et al. 2019; but see Berendzen et al. 2023). OXT can modulate social cognition at different levels. Enhanced OXT release in olfactory circuits increases social exploration and social recognition without interfering with other olfactory-dependent behaviors (Oettl et al. 2016). However, the modulation of social recognition by OXT subcutaneous injections follows an inverted U-shaped dose-response curve. Intermediate doses facilitate social recognition to a greater extent than low or high doses (Popik et al. 1992). In non-human primates, OXT boosts own- and other-regarding preferences (Chang et al. 2012), and in humans, OXT has been shown to promote social cognition and prosocial behavior, too (Jurek and Neumann 2018; Marsh et al. 2021). OXT in mice is also implicated in social learning (Dölen et al. 2013; Choe et al. 2015). For instance, systemic administration of OXT and vasopressin prolonged the memory recall of socially transmitted changes in drink preference (Popik and Van Ree 1993), suggesting OXT is indeed important for at least some cognitive aspects of STFP. However, direct evidence for the effects of OXT on STFP is, so far, elusive ( Lindeyer et al. 2013; but see Popik and Van Ree 1993).

In humans, OXT effects on social behavior have been shown to be subject to intergroup-biases: OXT promotes empathy, cooperation, trust and conformity with members of the same social group, but it fosters defensive behaviors and social distancing against members of a competing social group (De Dreu et al. 2010; Scheele et al. 2012; De Dreu and Kret 2016; Strang et al. 2017). Interestingly, in rodents, group affiliation seems to matter for social behavior, too. For instance, rats exhibit intergroup biases in prosociality (Ben-Ami Bartal et al. 2021), and there is evidence, although weak and inconclusive, that STFP also depends on the familiarity, i.e., group affiliation in a wider sense, between the observer and the demonstrator rat (Galef et al. 1984; Galef and Whiskin 2008a; Agee et al. 2019). It is therefore plausible to assume that any putative OXT effect on STFP might depend on the familiarity between demonstrator and observer.

In the current study, we therefore hypothesized that STFP in rats is modulated by OXT action, and that the predicted OXT effects on STFP are dependent on the familiarity between observer and demonstrator rats.

We trained rats in an adapted within-subject variant of the STFP paradigm (Galef and Whiskin 2008b; Jolles et al. 2011; Noguer-Calabús et al. 2022) that allowed us to quantify the individual magnitude in the change of socially transmitted food preference after relative to before social interaction. Briefly, observer rats reveal their original food preferences by choosing between two appetitive, differently flavored food rewards. Subsequently, they interact with a demonstrator rat who has been fed the food that was revealed non-preferred by the observer. After social interaction, we measure the observer rats’ food preferences again. Observers typically increase the consumption of the originally non-preferred pellets and/or decrease the consumption of the originally preferred pellets (Galef and Whiskin 2008b; Noguer‐Calabús et al. 2022).

We manipulated familiarity, as a proxy of group affiliation, between observers and demonstrators (Ben-Ami Bartal et al. 2014; Agee et al. 2019), as follows: during the social interaction phase of the STFP task, observers were either paired with a familiar cagemate demonstrator (in-group) or with an unfamiliar demonstrator from a different cage (out-group). To evaluate OXT effects on STFP, observers in the in-group and the out-group conditions received one of three treatments: vehicle injections, low-dose OXT, or large-dose OXT, systemically injected prior to social interaction. We measured the observers’ revealed food preferences before and immediately after social interaction, hence during acute OXT action, as well as one day later, when the exogenous OXT effects on the brain can be assumed to have faded. The second day of post-interaction preference testing allowed us to test whether OXT facilitates, or hampers, long-term social learning, and to rule out alternative explanations of putative changes in STFP.

## Materials and methods

### Subjects

We trained and tested 239 observer and 140 demonstrator Long-Evans male rats (Charles River, Germany) for this study, about 9–10 weeks old at arrival and weighing 410 ± 50 g on the injection day. 28 observers met the exclusion criteria (see below) and had to be removed from the analysis, leaving a final sample size of *n* = 211 observers. The temperature in the housing room was maintained at 22ºC ± 2ºC, with humidity set at 55% ± 2%. Subjects were kept under an inverted 12:12 light-dark cycle. Rats were supplied with laboratory rodent food (Sniff, Germany) and water ad libitum except for the STFP testing period when rats were food-restricted to 85% of their free-feeding body weight and fed daily after finishing the experimental procedure. All rats were handled for 5 min/day for 3 days before starting the experiment. All animal procedures were conducted in accordance with the German Welfare Act and were approved by the local authority LANUV (*Landesamt für Natur-*,* Umwelt- und Verbraucherschutz* North Rhine-Westphalia, Germany).

### Socially transmitted food preference task

#### Housing and habituation

Three days before the start of the STFP task, all rats underwent a 10-minute habituation session in an open field (50 × 50 × 45 cm, PVC, illumination to 5–15 lx). To this end, cagemates were placed together in the open fields. Upon habituation to the open field, all subjects were henceforth housed individually and were food-restricted. To habituate rats to the feeder setup, for three days, all rats were provided with hanging feeders in their home cages containing 10 grape-flavored and 10 banana-flavored pellets (TestDiet, USA). Then, rats were tested in the STFP task. The STFP protocol involved three stages: individual preference testing (days 1, 2, 3), social interaction (day 4), and post-interaction preference testing (days 4 and 5).

#### Individual preference testing

**Fig. 1:**
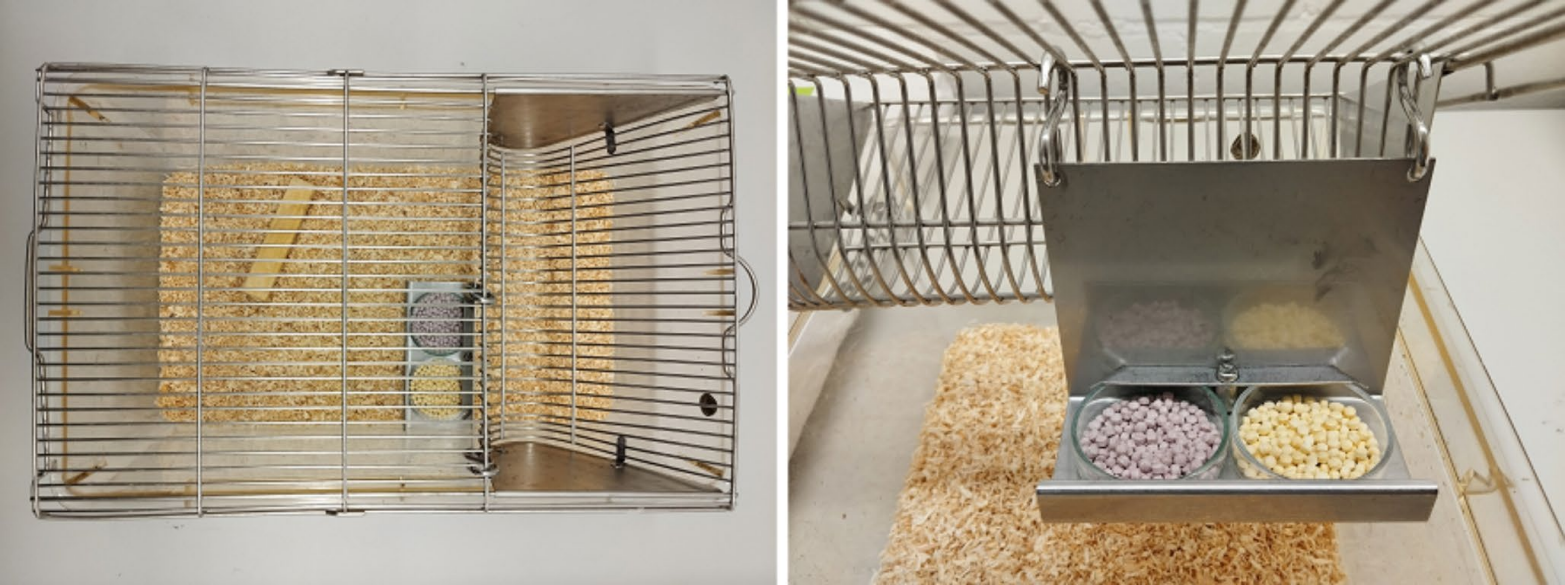
Photo example of the individual cage with the metal hanging feeder and two cups containing grape and banana pellets

On testing day one, observer rats were provided with two weighed cups, each of them containing a different pellet type (grape and banana). These cups were positioned in hanging feeders (pictured in Fig. 1), and observers had unrestricted access for 6 h. Subsequently, the cups were removed and weighed. This process was replicated over the next two days. The observers’ consumption was quantified individually and daily as the difference in cup weight before and after the 6-hour testing period. Upon concluding the pre-interaction testing, original individual preferences were determined by how much of each pellet type was consumed on day 3 (see exclusion criteria below).

#### Social interaction

On the fourth day of the STFP task, both observers and demonstrators were relocated to a room adjacent to the social interaction room. Demonstrators were fed with those pellets that were not revealed preferred on day 3 by their assigned observers. To enhance the corresponding odor, crushed pellets were spread to the demonstrator’s back, snout, and anal area. Then, demonstrators and observers were allowed to freely interact in the open field for 15 min. The interaction between the observer and demonstrator was recorded and an evaluator analyzed the time spent by the observer exploring the demonstrator using Solomon Coder (Solomon Coder beta 19.08.02 © András Péter).

#### Post-interaction preference testing

Following the interaction, observer rats were promptly returned to their individual cages and provided with two cups, each containing one of the two banana- and grape-flavored food types. Similar to the pre-interaction testing, the cups were taken out and weighed after a 6-hour interval. The same preference test was repeated the next day. Subsequently, all animals were reintegrated into prior group housing.

#### Exclusion criteria

If a rat revealed preferred a particular pellet type on day 3 that was different from the pellet type revealed preferred on days 1 *and* 2, we assumed that this rat’s preferences were inconsistent since it was not evidently clear what the truly preferred reward was on day 3. Rats with inconsistent preferences were excluded from further analysis. For example, if a rat preferred grape pellets on days 1 *and* 2, but banana pellets on day 3, it would be excluded from analysis since we could not tell with certainty if this rat truly preferred banana, or grape. The reason for this exclusion criterion is to make sure that demonstrators were fed with the truly non-preferred food, and to avoid accidentally feeding the demonstrator with actually preferred food.

### Familiarity group assignment

We operationalized group affiliation as familiarity between observers and demonstrators (Ben-Ami Bartal et al. 2014). Therefore, there were two familiarity groups: the in-group and the out-group. In the in-group condition, pairs of observers and demonstrators (*n* = 100) were housed together in one cage upon arrival at the animal housing. In the out-group condition, pairs of observers were housed together, but in separate cages from the demonstrators (three demonstrators per cage) to prevent contact before the STFP interaction. The out-group consisted of 111 observers and 40 demonstrators. In general, rats were housed according to this group assignment protocol for 2–3 weeks upon arrival in the animal facility; at the start of the experiment, they were housed individually (see below).

### Oxytocin treatment

Within each familiarity group, observers were randomly assigned to one of three treatment groups: the control group (vehicle = saline), the group treated with low-dose OXT (0.25 mg OXT/ml), and the large-dose OXT group (1.0 mg OXT/ml), with an injection volume of 1 ml/kg. All observers received a single intraperitoneal injection immediately before the social interaction phase during the STFP.

### Data analysis

We used a mixed analysis of variance (ANOVA; SPSS 27.0.1, IBM, USA; R 4.0.2; R Core Team, 2020, special usage of the ggbreak package for plotting (Xu et al. 2021) with the dependent variable *pellet consumption* (grams eaten), and the within-subject factors *pellet preference* (originally preferred vs. non-preferred pellets), *day* (pre-interaction day 3 vs. post-interaction day 4 vs. post-interaction day 5), and the between-subject factors *familiarity* (in-group vs. out-group) and *treatment* (vehicle vs. low-dose OXT vs. large-dose OXT). Post hoc analyses were performed with two-sided t-tests. Benjamini–Hochberg correction was applied to correct for multiple comparisons.

Occasionally, rats exhibited very strong STFP, resulting in a full preference reversal post- vs. pre-interaction. Preference reversals were defined as higher consumption of the originally non-preferred food than the originally preferred food after the social interaction on day 4. We compared the frequency of full preference reversals between conditions with a Fisher’s exact test.

Finally, we measured the time the observer spent socially exploring the demonstrator during the social interaction phase of the STFP. We examined observer behavior exclusively because existing literature indicates minimal effects of the demonstrator’s behavior on the observers’ STFP performance (Galef and Wigmore 1983; Galef and Whiskin 2008a). To detect differences in social interaction times between groups and conditions, we employed a mixed ANOVA and its corresponding post-hoc two-sided t-tests and corrections for multiple comparisons.

## Results

### Familiarity modulates STFP in vehicle rats

**Fig. 2:**
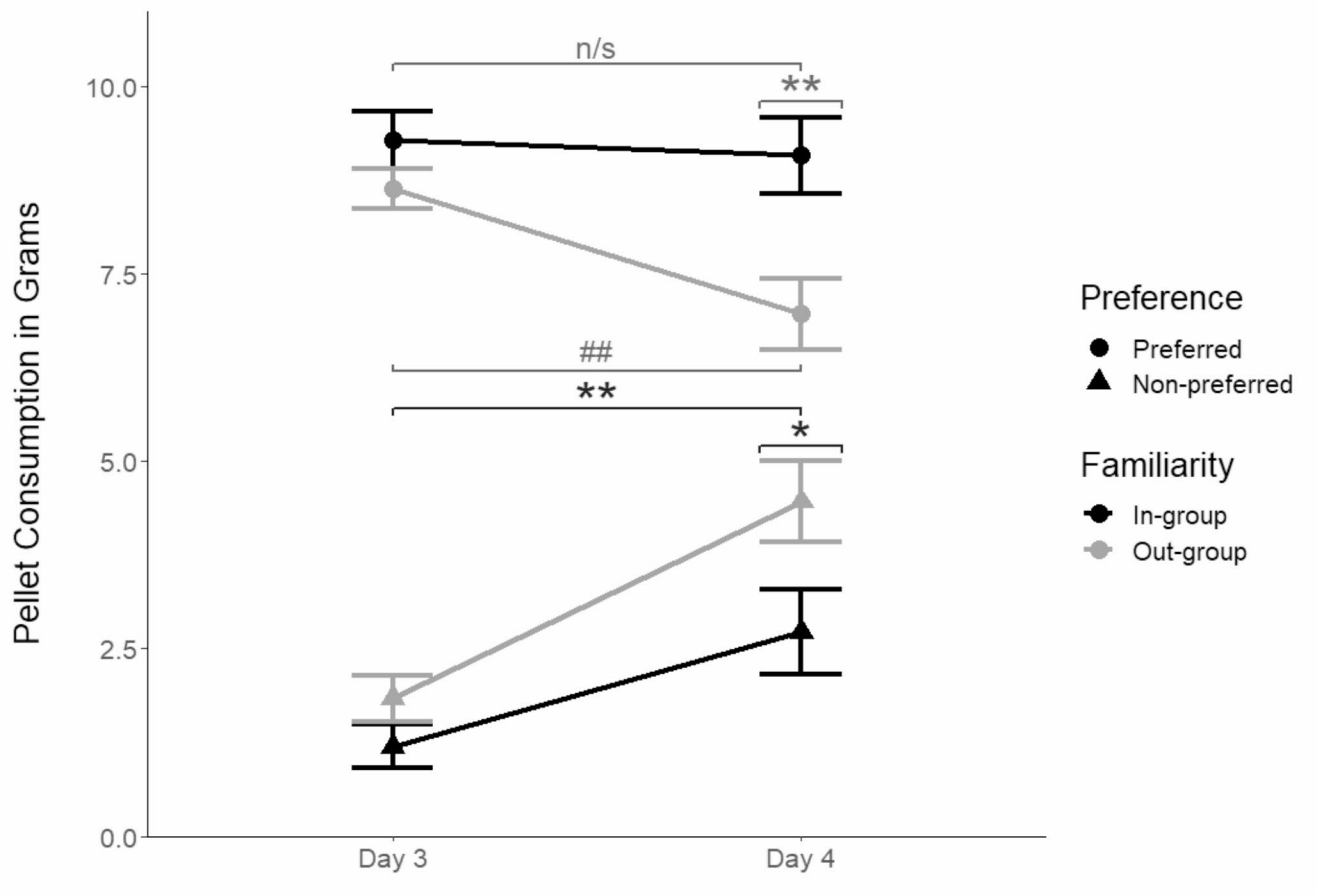
Vehicle-treated observers’ socially transmitted food preferences are modulated by familiarity. Mean (± standard error of the mean; SEM) of the pellets (originally preferred, circle; originally non-preferred, triangle) consumed on days 3 (pre-social interaction) and day 4 (post-social interaction) by observers who interacted with a familiar demonstrator (in-group (*n* = 31), black) or an unfamiliar one (out-group (*n* = 40), light gray). The change in consumption of the originally non-preferred pellets pre- vs. post-interaction was stronger in the out-group than the in-group, and a change in consumption of the originally preferred pellets was only found in the out-group. * *p* < .05; ** *p* < .01; ## out-group *p* < .01, n/s in-group *p* > .05

To evaluate how familiarity modulates STFP in general, i.e., in the absence of OXT effects, we compared the amount of pellets eaten by vehicle observers between days 3 and 4, i.e., before vs. immediately after social interaction, as a function of familiarity (in- vs. out-group) and pellet preference (originally preferred vs. non-preferred pellets; Fig. 2). The mixed ANOVA showed a simple main effect of pellet preference on amount consumed (*F*_[1, 67]_ = 136.816, *p* = .000) and a simple main effect of day (*F*_[1, 67]_ = 23.969, *p* = .000), as well as an interaction effect between pellet preference and day (*F*_[1, 67]_ = 21.837, *p* = .000), suggesting that rats showed STFP. Importantly, we also found a significant interaction effect between pellet preference and familiarity (*F*_[1, 67]_ = 8.405, *p* = .005). The post-hoc tests (all post-hoc tests were corrected for multiple comparisons) indicated that both familiarity groups increased their consumption of the originally non-preferred pellets on day 4 compared to day 3 (in-group: *t*_[30]_ = -3.16, *p* = .005; out-group: *t*_[37]_ = -5.53, *p* = .000), suggesting that STFP was found in both familiarity groups. However, a between-group comparison showed that consumption of the originally non-preferred pellets was higher in the out-group than the in-group on day 4 (in- vs. out-groups: *t*_[66.2]_ = -2.19, *p* = .032), implying stronger STFP in the out-group than the in-group. Consistent with this conclusion, only the out-group decreased the consumption of their originally preferred pellets on day 4 compared to day 3 (out-group: *t*_[37]_ = 3.14, *p* = .005; in-group: *t*_[37]_ = 0.48, *p* = .635) and compared to the in-group (day 4 in- vs. out-groups: *t*_[65.7]_ = 3, *p* = .004). Accordingly, the change in consumption of the originally preferred pellets, but not non-preferred pellets, from day 3 to 4 differed between familiarity groups (difference in originally preferred pellets: *t*_[66.9]_ = -2.05, *p* = .044; originally non-preferred pellets: *t*_[65.7]_ = 1.56, *p* = .124). Hence, both familiarity groups exhibited socially transmitted food preferences, but the effect was significantly more pronounced in the out-group than the in-group (Fig. 2).

#### Full preference reversal

**Fig. 3:**
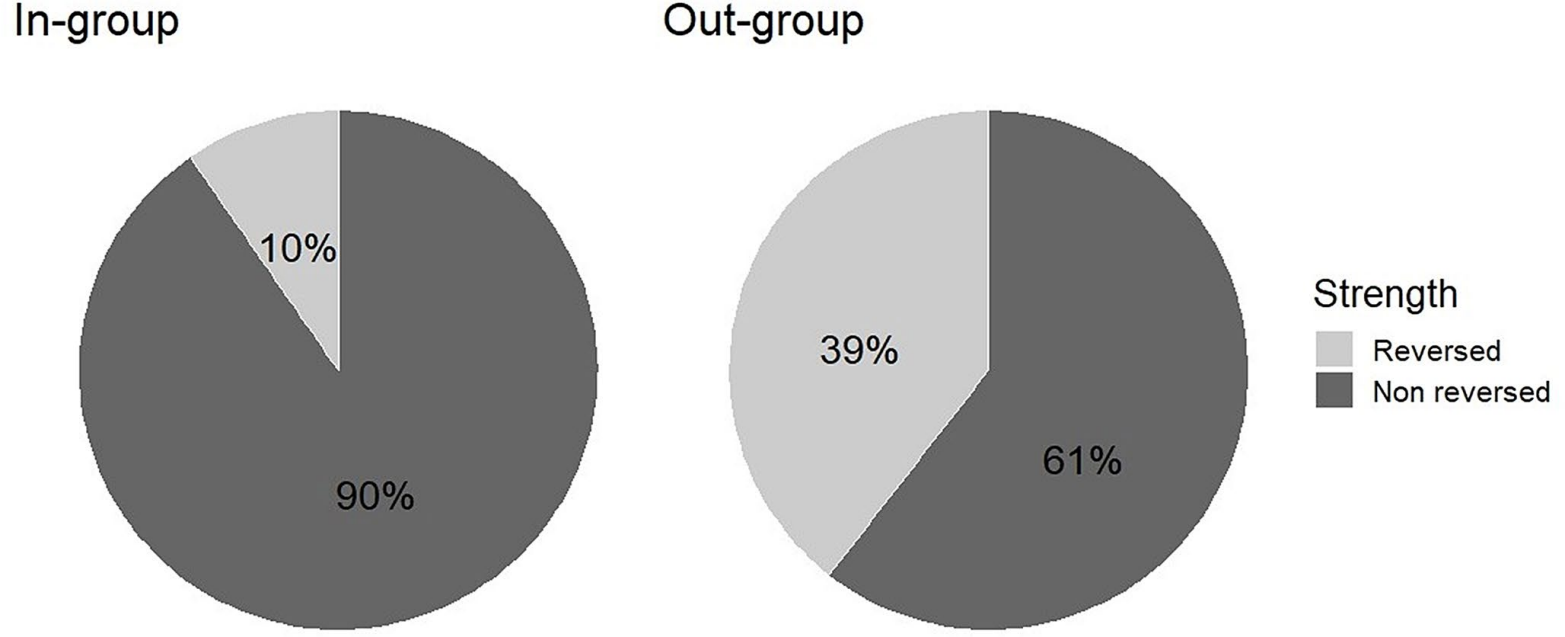
Frequency of full preference reversals, in percent, after social interaction (day 4 vs. day 3). The frequency of full preference reversals was significantly higher in the out-group than in the in-group

Rats occasionally exhibited very strong STFP, resulting in a full preference reversal on day 4 vs. day 3. We computed the proportion of vehicle-treated observers who fully reversed their pellet preferences, and compared the proportion of pellet preference reversals between familiarity groups (Fig. 3). In the in-group, only 10% of rats (3/31) fully reversed their pellet preferences, in contrast to the out-group, where 39% of rats (15/38) did so. Hence, consistent with the conclusion of the previous paragraph, these data suggest stronger social transmission of food preferences in the out-group than the in-group condition (Fisher’s exact test; *p* = .006, two-sided). Further analyses are available in the supplemental materials, Fig. 1.

### Oxytocin effects on social transmission of food preference are modulated by familiarity

To find out if the OXT treatment had an effect on STFP, possibly in a familiarity-dependent way, we ran a four-way mixed ANOVA with pellet preference (originally preferred vs. non-preferred), familiarity (in- vs. out-group), treatment (vehicle vs. low-dose OXT vs. large-dose OXT), and day (days 3 vs. 4 vs. 5) as independent variables on pellet consumption. We found a significant simple main effect of pellet preference (*F*_[1, 202]_ = 440.333, *p* = .000), a significant simple main effect of treatment (*F*_[2, 202]_ = 9.079, *p* = .000), and a significant simple main effect of day (*F*_[2, 404]_ = 16.129, *p* = .000), and a significant four-way interaction between pellet preference, familiarity, treatment and day (*F*_[3.51, 354.66]_ = 3.029, *p* = .023).

**Fig. 4:**
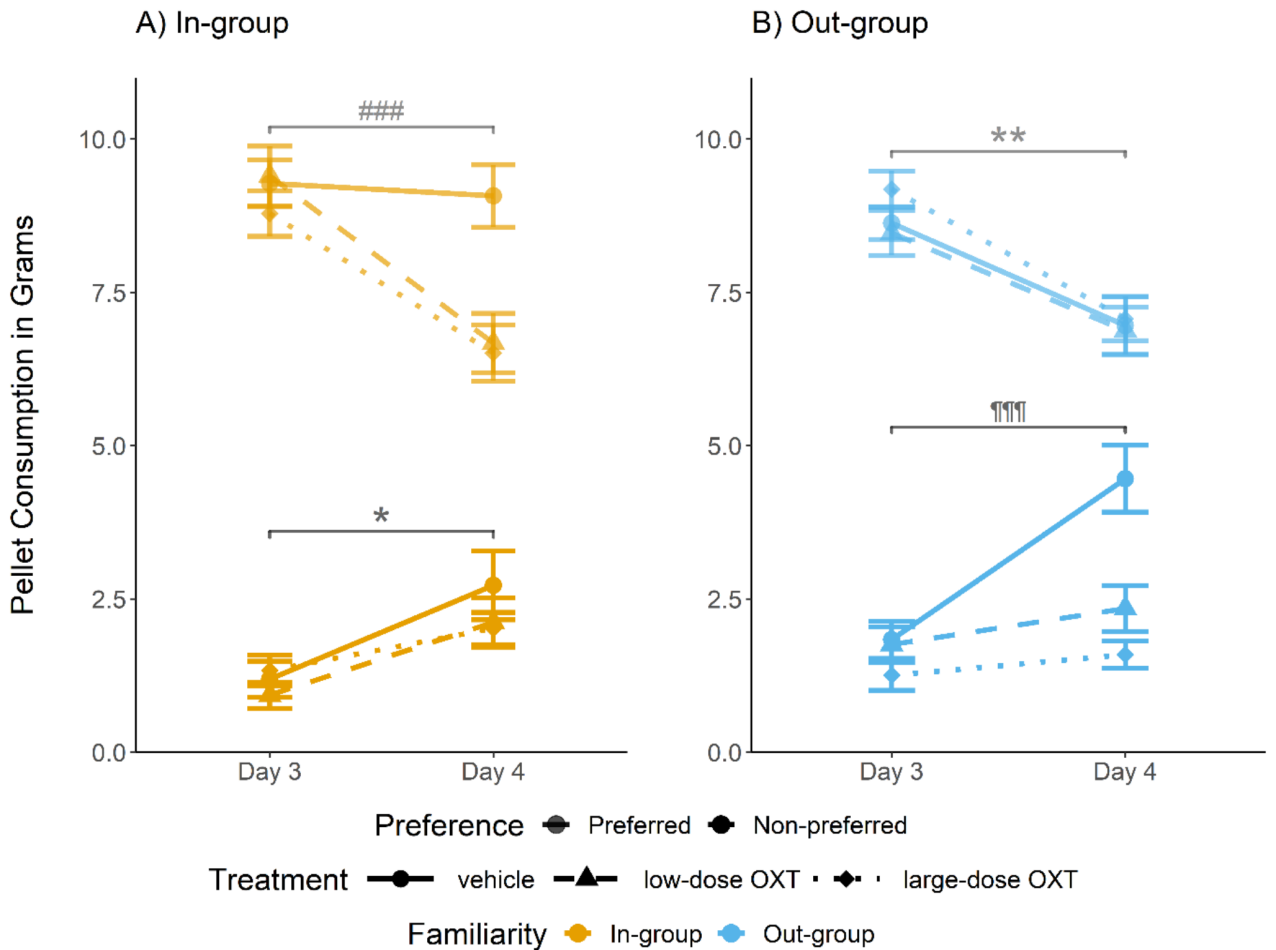
Acute oxytocin (OXT) and intergroup effects on socially transmitted food preference (STFP). STFP in the in-group (panel A), and the out-group (panel B). In both panels, the pellet consumption (mean ± SEM) of the vehicle group is represented by the solid line and circle symbols, the low-dose OXT group by the dashed line and triangles, and the large-dose OXT group by the dotted line and squared symbols. The originally preferred pellets (upper lines) are indicated in a slightly transparent hue, and the originally non-preferred pellets (lower lines) are in an opaque hue. In the in-group (panel A), rats in all treatment conditions increased their consumption of the originally non-preferred pellets after social interaction on day 4, thus exhibiting STFP. Unlike rats in the vehicle group, rats that received OXT injections prior to social interaction decreased the consumption of the originally preferred pellets. In the out-group (panel B), OXT administration prevented the increased consumption of the originally non-preferred pellets observed in the vehicle group, thus blocking STFP. However, there were no differences between treatment conditions in the consumption of the originally preferred pellets, which decreased between days 3 and 4. * All treatments *p* < .05; ** all treatments *p* < .01; ### OXT-treated groups *p* < .001; ¶¶¶ vehicle group *p* < .001

To unpack this complex interaction effect, we ran a suite of post-hoc tests (again, all post-hoc tests were corrected for multiple comparisons). To understand the acute effects of OXT on STFP, we, first, zoomed in on what happened on day 3 vs. day 4 (Fig. 4; individual data plots in Fig. 2 in the supplemental materials). In the in-group (panel A of Fig. 4), we found a significant decrease in consumption of the originally preferred pellets on day 3 vs. day 4 in both OXT groups (low-dose OXT: *t*_[32]_ = 5.69, *p* = .000; large-dose OXT: *t*_[35]_ = 6.69, *p* = .000), but not in the vehicle group (*t*_[30]_ = 0.48, *p* = .714). There was a significant increase in consumption of the originally non-preferred pellets from day 3 to day 4 in all treatment groups (vehicle: *t*_[30]_ = -3.16, *p* = .01; low-dose OXT: *t*_[31]_ = -2.93, *p* = .013; large-dose OXT: *t*_[35]_ = -3.4, *p* = .007), and we found no significant difference in their consumption over days 3 and 4 between treatment groups (vehicle vs. low-dose OXT: *t*_[59.4]_ = -0.476, *p* = .636; vehicle vs. large-dose OXT: *t*_[40.4]_ = -1.61, *p* = .232; low-dose OXT vs. large-dose OXT: *t*_[45]_ = -1.16, *p* = .38). This analysis suggests that, in the in-group, OXT administration led to a stronger decrease in consumption of the originally preferred pellets relative to vehicle administration, but had no marked effect on the consumption of the originally non-preferred pellets.

The picture was different in the out-group (panel B of Fig. 4). Here, we found a significant decrease in consumption of the originally preferred pellets on day 3 vs. day 4 in all treatment groups, including the vehicle group (vehicle: *t*_[37]_ = 3.14, *p* = .006; low-dose OXT: *t*_[33]_ = 3.82, *p* = .002; large-dose OXT: *t*_[36]_ = 6.14, *p* = .000). There was no significant difference in the change in consumption of the originally preferred pellets between any of the treatment groups (vehicle vs. low-dose OXT: *t*_[69.3]_ = -0.087, *p* = .931; vehicle vs. large-dose OXT: *t*_[66.2]_ = -0.97, *p* = .504; low-dose OXT vs. large-dose OXT: *t*_[65.5]_ = -0.969, *p* = .504). By contrast, we found a significant and steep increase in consumption of the originally non-preferred pellets from day 3 to day 4 in the vehicle group (*t*_[37]_ = -5.53, *p* = .000), but no significant increase in either OXT group (low-dose OXT: *t*_[33]_ = -2.12, *p* = .061; large-dose OXT: *t*_[36]_ = -1.7, *p* = .136). Accordingly, vehicle observers in the out-group condition consumed significantly more of the originally non-preferred pellets than the OXT-treated observers (vehicle vs. low-dose OXT: *t*_[63.3]_ = 3.15, *p* = .024; vehicle vs. large-dose OXT: *t*_[48.4]_ = 4.77, *p* = .000; low-dose OXT vs. large-dose OXT: *t*_[54.3]_ = 1.74, *p* = .226). This analysis suggests that in the out-group, OXT had different effects on STFP than in the in-group. In the out-group condition, relative to vehicle administration, OXT dampened the increase in consumption of the originally non-preferred pellets, but it had no marked effect on the consumption of the originally preferred pellets.

### Oxytocin has long-term effects on social transmission of food preferences

**Fig. 5:**
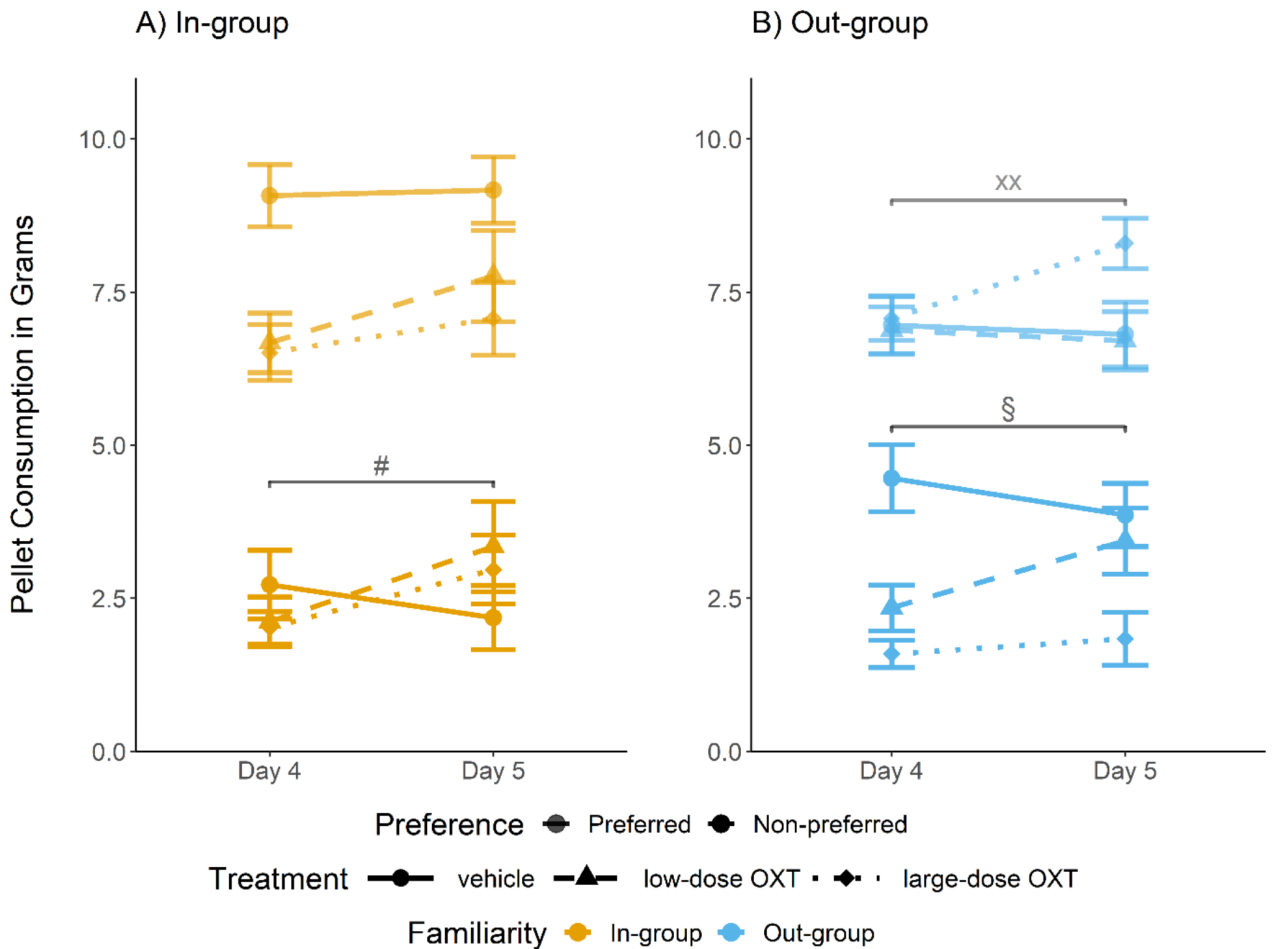
Long-term oxytocin (OXT) and familiarity effects on socially transmitted food preference (STFP). Line and panel representations are the same as Fig. 4. In the in-group (panel A), rats treated with OXT on day 4 (immediately after social interaction and OXT injection) increased the consumption of the originally non-preferred pellets on day 5 (one day after social interaction and OXT injection) following the previous tendency (from day 3 to day 4). By contrast, the consumption of the vehicle group was relatively constant across days. The consumption of the originally preferred pellets was constant for all treatment conditions. In the out-group (panel B), only the low-dose OXT group increased the consumption of the originally non-preferred pellets from day 4 to day 5. By contrast, the OXT effects on the large-dose OXT group were long-lasting, suggesting that the OXT-related blocking of STFP was stable over time. Regarding the originally preferred pellets, only the large-dose OXT group increased their consumption once acute OXT effects dissipated on day 5; the other treatment conditions remained unchanged. # OXT-treated groups *p* < .05; § low-dose OXT group *p* < .05; XX large-dose OXT group *p* < .05

So far, we presented the results of day 3 (before social interaction) vs. day 4 (immediately after social interaction and immediately after OXT injection, i.e., with acute OXT effects on the rats’ system). To understand if OXT had long-term effects on STFP (Fig. 5; individual data plots in Fig. 3 in the supplemental materials), beyond its acute action, we extended our post-hoc analysis to day 5, i.e., one day after OXT or vehicle injection. In the in-group (panel A of Fig. 5), there was no significant difference in originally preferred pellets consumption between day 4 and day 5 in any of the treatment groups (vehicle: *t*_[30]_ = -0.222, *p* = .826; low-dose OXT: *t*_[32]_ = -1.9, *p* = .091; large-dose OXT: *t*_[35]_ = -1.22, *p* = .292). By contrast, both OXT groups, but not the vehicle group, showed a continued increase in consumption of the originally non-preferred pellets from day 4 to day 5 (vehicle: *t*_[30]_ = 1.19, *p* = .292; low-dose OXT: *t*_[32]_ = -2.38, *p* = .043; large-dose OXT: *t*_[35]_ = -2.26, *p* = .045), even though the amount of originally non-preferred pellets consumed on day 5 did not differ between OXT and vehicle groups (vehicle vs. low-dose OXT: *t*_[57]_ = -1.28, *p* = .635; vehicle vs. large-dose OXT: *t*_[65]_ = -1.02, *p* = .635; low-dose OXT vs. large-dose OXT: *t*_[61.1]_ = 0.404, *p* = .843). Hence, in the in-group, the pattern of effects on STFP observed under acute OXT effects (day 4) persisted, or even increased, on day 5, when the acute OXT effects on the organism can be assumed to have waned.

In the out-group (panel B of Fig. 5), we found a significant increase in consumption of the originally preferred pellets in the large-dose OXT group from day 4 to day 5, but not in the low-dose OXT or vehicle groups (vehicle: *t*_[37]_ = 0.944, *p* = .395; low-dose OXT: *t*_[33]_ = 0.366, *p* = .717; large-dose OXT: *t*_[36]_ = -3.35, *p* = .004). Although we had found significant OXT effects on the consumption of the originally non-preferred pellets on day 4 (see above), this difference disappeared on day 5 for the low-dose OXT (vehicle vs. low-dose OXT: t_[70.7]_ = 0.565, *p* = .861) and only remained significant for the large-dose OXT (vehicle vs. large-dose OXT: t_[73.6]_ = 3.01, *p* = .024; low-dose OXT vs. large-dose OXT: t_[64.3]_ = 2.31, *p* = .086). In line with this observation, the low-dose OXT group showed an increase in consumption of the originally non-preferred pellets from day 4 to day 5 (*t*_[33]_ = -2.45, *p* = .033), but, the large-dose OXT group continued to show no significant change in consumption of the originally non-preferred pellets from day 4 to 5 (*t*_[36]_ = -0.608, *p* = .579), suggesting that they never acquired STFP. In sum, also in the out-group, we found a complex pattern of results suggestive of the fact that the effects of OXT on STFP outlasted its acute action. Hence, overall, our results suggest that OXT effects on STFP were dependent on familiarity with the demonstrator and reflect long-lasting changes in social learning.

### Familiarity-modulated OXT effects on STFP cannot be explained by anorexic effects or social exploration time

Acute OXT action has anorexic effects, especially on palatable food (Olszewski et al. 2010; Herisson et al. 2014). It is therefore possible that the complex pattern of OXT effects on STFP reported here can simply be explained by its anorexic effects. Indeed, we found that OXT injections decreased total pellet consumption (originally preferred and non-preferred pellet types combined) on the day of injections (mixed ANOVA with the factors familiarity, treatment and day; main effect of treatment, *F*_[2, 202]_ = 9.079, *p* = .000; significant simple main effect of day, *F*_[2, 404]_ = 16.129, *p* = .000, and a significant interaction effect between treatment and day, *F*_[4, 404]_ = 21.241, *p* = .000, Fig. 4 supplemental material). However, these anorexic effects were restricted to day 4, i.e., the day of OXT injection, and did not extend to day 5 (post-hoc test: day 3 vs. day 5; in- low-dose OXT: *t*_[31]_ = -1.6, *p* = .165; in- large-dose OXT: *t*_[35]_ = 0.276, *p* = .802; out- low-dose OXT: *t*_[33]_ = 0.252, *p* = .802; out- large-dose OXT: *t*_[36]_ = 1.25, *p* = .284). In addition, even though we found group-dependent OXT effects on STFP (see analysis above), OXT effects on total pellet consumption did not differ between in-group and out-group (*F*_[1, 202]_ = 0.352, *p* = .554). Our analysis presented above showed that OXT effects on STFP were group-dependent and long-lasting, but OXT effects on total pellet consumption were neither group-dependent, nor long-lasting, suggesting that the reported OXT effects on STFP cannot be straightforwardly explained by its anorexic effects (Table 1 supplemental material; see discussion for further elaboration).

In addition to OXT anorexic effects, OXT and/or familiarity may have modulated the time observers spent interacting with, or socially exploring, the demonstrators. A mixed ANOVA revealed a significant simple main effect of OXT treatment, but not familiarity, on social exploration time (treatment: *F*_[2, 224]_ = 7.247, *p* = .000; familiarity: *F*_[1, 224]_ = 1.364, *p* = .244): rats treated with the large-dose of OXT explored the demonstrators less than the other treatment groups (vehicle vs. low-dose OXT: *t*_[149]_ = 0.763, *p* = .447; vehicle vs. large-dose OXT: *t*_[145]_ = 3.55, *p* = .000; low-dose OXT vs. large-dose OXT: *t*_[148]_ = 2.7, *p* = .012; Fig. 5 supplemental material). Even though the observation that the demonstrator’s novelty in the out-group, relative to the in-group, did not lead to a significantly longer duration of partner exploration is somewhat surprising (Oettl et al. 2016), the lack of evidence for a difference in social exploration time suggests that exploration time unlikely explains the familiarity effects on STFP reported above. Likewise, although we did find OXT effects on exploration time, we did not find a significant interaction between OXT and familiarity, suggesting that the complex interaction of OXT and familiarity on STFP cannot be explained by social exploration.

## Discussion

In this study, we measured the effects of systemic injections of OXT and the familiarity between observer and demonstrator on STFP. First, our results showed that vehicle rats revealed stronger changes in food preference when encountering an unfamiliar than a familiar demonstrator. Second, we found that systemic OXT administration influenced STFP dependent on whether the demonstrator was familiar or not: when the demonstrator was familiar (in-group), OXT led to a decreased consumption of the originally preferred pellets after social interaction with the demonstrator, but had no effect on the consumption of the originally non-preferred pellets. By contrast, we found opposite effects of OXT on STFP when the demonstrator was unfamiliar (out-group): OXT, relative to vehicle, did not change the consumption of the originally preferred pellets, but, notably, prevented the increase in consumption of the originally non-preferred pellets. These familiarity-dependent OXT effects on STFP could still be found one day later, at least after large OXT doses, when the acute effects of OXT on the organism most likely had waned, suggesting that OXT action during social interaction has long-term effects on STFP. Our results uncover a new mechanism how OXT modulates familiarity-dependent socially transmitted preferences and social reward revaluation.

Previous literature identified an acute anorexic effect after OXT administration in male rats, resulting in less food consumption (Arletti et al. 1989, 1990; Benelli et al. 1991). Our results also show a decrease in total pellet consumption (originally preferred + non-preferred) by the OXT groups. Although anorexic effects might explain our pattern of results, we believe this is not the case. First, if OXT’s anorexic effects were the only mechanism, it should reduce consumption of both preferred and non-preferred pellets equally, but we did not find this to be the case (see results above). Second, OXT effects depended on the demonstrator’s familiarity – an observation that is also difficult to reconcile with the anorexia hypothesis. Third, and perhaps most importantly, we found that OXT effects on STFP outlasted the acute OXT effects on total pellet consumption, suggesting that OXT action had long-lasting effects on STFP beyond its acute anorexic effects. We, hence, conclude that the results reflect group-dependent OXT effects on social learning, and not merely an OXT-related reduction in hunger or appetite.

Can the observation that vehicle rats showed stronger STFP with unfamiliar than familiar demonstrators be explained by differences in social exploration times? A feasible explanation of this phenomenon in rats is their preference for social novelty. Rats typically interact longer with an unfamiliar individual, which could enhance the chance of olfactory transmission of the demonstrator’s food preference via its breath (Galef et al. 1988; Galef and Whiskin 2008a). However, a more recent study could not find support for this explanation, as a more detailed analysis showed that observers spent equal time sniffing the face of their demonstrator or in direct nose contact regardless of familiarity (Agee et al. 2019). In agreement with that, our vehicle rats in both in- and out-group conditions spent equal time sniffing their demonstrator, suggesting that other mechanisms than merely olfactory recognition or social interaction time accounted for STFP.

So, how can we explain the familiarity- and OXT-dependent changes in consumption of the originally preferred and originally non-preferred pellets? One possibility is that OXT affected the decision weight the observers’ placed on the specific kind of social information transmitted by the demonstrator in a familiary-dependent way: in the out-group condition, unlike the control observers, OXT-treated observers simply ignored the food information that was socially transmitted by the demonstrator, and, hence, continued to consume their originally preferred pellets the same way as they did before the social interaction. By contrast, in the in-group condition, OXT-treated observers began to dislike the pellets that were not eaten by the demonstrator, and, consequently, reduced the consumption of those pellets.

However, there are alternative explanations for the complex familiarity- and OXT-dependent effects on STFP that seem equally plausible. For example, one could argue that the information that is transmitted by the demonstrator in STFP would be the palatability of the originally non-preferred reward, but there would be no information transmitted about the originally preferred reward; after all, observer rats smell the scent of the originally non-preferred reward in the demonstrators’ breath (Galef et al. 1988), but do not have any social information on the originally preferred pellets. Hence, STFP would mainly manifest as an increase in consumption of the originally non-preferred reward. Since, in vehicle rats, total pellet intake (preferred + non-preferred pellets) usually remains constant after social interaction, the decrease in consumption of the originally preferred reward in STFP would just be the logical, secondary consequence of the increased consumption of the originally non-preferred rewards: if rats eat more of food B after social interaction, they necessarily have to eat less of food A, unless they change their total food intake. According to this view, the difference in consumption of the originally preferred pellets between OXT and vehicle rats in the in-group might just reflect a secondary satiation effect: as mentioned, OXT led to a decreased total amount of pellets eaten on day 4, after the social interaction (see results and supplemental material). OXT-treated rats in the in-group showed STFP much like the vehicle rats, and accordingly ramped up their consumption of the originally non-preferred pellets (Fig. 4), while, at the same time, reducing their overall pellet consumption due to OXT’s anorexic action. Hence, the OXT-related decrease in consumption of the originally preferred pellets on day 4 (Fig. 4) may simply reflect satiation effects: [reduced total consumption] minus [increased non-preferred consumption] = [reduced preferred consumption]. Note that this explanation may account for the pattern of results found in the in-group results, but cannot account for our out-group results. Future research needs to disentangle whether the familiarity- and OXT-dependent changes in pellet consumption reported here reflect familiarity-dependent differences in the decision weights attached to social information about the preferred and the non-preferred rewards, or differential satiation effects.

OXT’s role in diverse modes of social information processing has become a focus of emerging research, making it a strong candidate for regulating social transmission of food value (Popik and Van Ree 1993; Choleris et al. 2009; but see Lindeyer et al. 2013). A study demonstrated the pivotal role of centrally released OXT in social cue processing, which integrates both odor extraction and social recognition. OXT affected genuine social aspects of social cue processing, as evidenced by the fact that inhibiting OXT signaling in the anterior olfactory nucleus (AON) resulted in compromised social recognition, while object and non-social odor recognition abilities remained unaffected (Oettl et al. 2016). In agreement with the notion that OXT facilitates the olfactory detection of information transmitted by a conspecific, further studies elaborated on that topic. It was shown that OXT signalling in the olfactory sensory cortex is crucial for the association between neutral odors and socially meaningful cues (Choe et al. 2015). Even more strikingly, meeting a conspecific differing in either age or sex activated discrete patterns of OXT neurons in the lateral septum and/or medial amygdala in male rats, hinting at independent subcircuits for certain social modalities (Lukas et al. 2013). While these findings do not explicitly address the different familiarity-dependent OXT effects on STFP in the in- and out-group conditions reported here, it may be reasonable to assume that demonstrators’ familiarity, too, activates specialized OXT subcircuits, explaining our observed intergroup differences in flexible social preference revaluation. The differences in STFP between the in-group and out-group conditions might also be suggestive of familiarity effects on the recollection success of social reward revaluation. This familiarity-moderated recollection of reward value might involve hippocampal circuits as they are necessary for STFP (Alvarez et al. 2001; Winocur et al. 2001; Clark et al. 2002; but see Burton et al. 2000; Thapa et al. 2014) though selective OXT effects on GABA action in hippocampus (Maniezzi et al. 2019).

In conclusion, the current study provides evidence that STFP is modulated by OXT in a familiarity and dose-dependent manner. While the socially transmitted changes in food preference were stronger when interacting with strangers, large OXT dosage blocked the integration of social information during reward revaluation. The presented study is in line with the current understanding that OXT can modulate sensitivity to socially significant cues. The interpretation of these cues is affected by contextual elements, particularly the familiarity of the demonstrator, suggesting that OXT has social effects beyond facilitating prosocial behavior (Anacker and Beery 2013; Olff et al. 2013; Love 2014; Piva and Chang 2018). These results add a layer of complexity to our knowledge of the influence of OXT in social learning. Exploring responsible neuronal areas and their specific dependency requires further investigation.

## Electronic supplementary material

Below is the link to the electronic supplementary material.

Supplementary Material 1

## Data Availability

Raw data supporting the findings presented in the study is openly available in OSF at https://osf.io/sfg3x/.
