# Supplementary material for: Oxytocin effects on socially transmitted food preferences are moderated by familiarity between rats
Source: Psychopharmacology (Berl). 2024 Sep 25;242(2):361–72. doi: 10.1007/s00213-024-06682-x (PMC11775072; doi:10.1007/s00213-024-06682-x)
Supplement: Supplementary file 1 — Supplementary Material 1 [file 213_2024_6682_MOESM1_ESM.docx]

**SUPPLEMENTAL MATERIAL**

**Oxytocin effects on socially transmitted food preferences are moderated by familiarity between rats**

Irina Noguer-Calabús^a^, Sandra Schäble^a^, José Dören^a^, Tobias Kalenscher^a^

^a^Comparative Psychology, Institute of Experimental Psychology, Heinrich-Heine University Düsseldorf, Universitätsstraße 1, 40225 Düsseldorf, Germany

Corresponding author: noguerca@hhu.de

Conflict of interest statement: The authors declare no competing interests.

Acknowledgment: The project was supported by a grant from the German Research Foundation (Deutsche Forschungsgemeinschaft, DFG, grant no. KA 2675/5-3) to TK

**Reward preference strength before social interaction predicts the probability of preference reversal on the day of social interaction (day 4)**

In supplemental analyses, we asked whether the likelihood of preference reversals on day 4 depended on how strongly rats preferred one reward over the other on day 3. To this end, we first calculated a Preference Index (PI) which expresses the preference strength for one type of pellets over another on a given test day. A PI value of 1 indicates that the rat exclusively eats pellet type A, 0 indicates equal consumption of both pellet types (reward indifference), and -1 means the rat consumes only the other pellet type B. The PI is based on the grams (g) of each pellet type A and B consumed on a given day, using the following equation:

$${PI}_{Day}=\frac{\left( {Pellet}_{A}\left( g \right)-{Pellet}_{B}(g) \right)}{\left( {Pellet}_{A}\left( g \right)+{Pellet}_{B}(g) \right)}$$

We computed a PI for each day and rat, and compared the PIs of day 4 with those of day 3. If a rat reversed its preference on day 4 (eating more originally non-preferred pellets than originally preferred), rats were categorized into the “reversal” group, the other rats were categorized into the “non_reserval” group.

A general linear model showed that the likelihood of full preference reversals depended on the preference strength at day 3 for the originally preferred pellet type: the weaker the original preference before the social interaction, as expressed by PIs closer to 0, the more likely a full preference reversal after social interaction (Fig. 1; preference strength day 3: *z* = -3.522; *p* = 0.000428; familiarity group: *z* = 0.456; *p* = 0.648252; interaction between preference strength day 3 and familiarity: *z* = 0.063; *p* = 0.949587). Therefore, rats with a stronger preference before the social interaction were less likely to fully reverse their food preferences after social interaction, independently of the familiarity with the demonstrator. Importantly, the original preference strength before social interaction was not different between the in-group and the out-group ( *t*_[208]_ = 1.61, *p* = .11), suggesting that the familiarity effect on preference reversals was independent of the original preference strength effect.


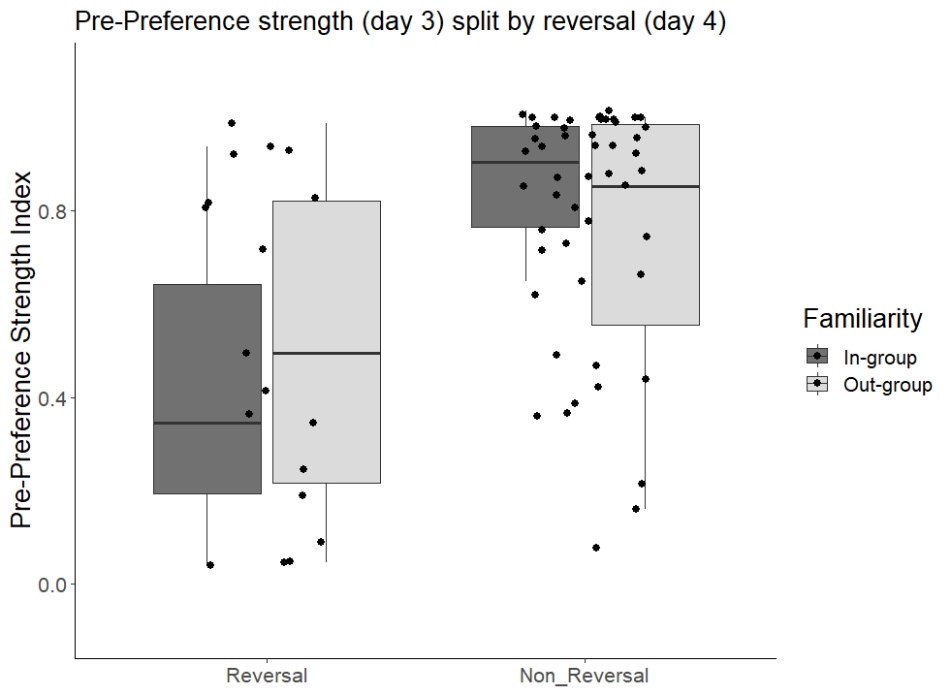


**Fig. 1** Pre-preference strength, i.e., the strength of preference for one reward over the other before social interaction on day 3 as expressed by the preference index PI (see text), predicted the likelihood of full preference reversals on day 4. The horizontal line inside the boxes represents the median preference index on day 3, the hinges represent the 25^th^ and 75^th^ percentiles, and the whiskers represent the interquartile ranges. Dots represent individual data points. The preference strength on day 3 was higher for the observers that did not reverse their food preferences on day 4 than for the ones that did so, independently of familiarity with the demonstrator.

**Acute oxytocin (OXT) effects on STFP: Individual data plots**

Individual data plots can reveal important information that can be masked when plotting the averages of the STFP results. Therefore, here, we display the individual choice behavior of the rats on days 3 (pre-social interaction) and day 4 (first post-social interaction testing day) for each familiarity group (in- vs. out-group), treatment condition (vehicle vs. low-dose OXT vs. large-dose OXT), and pellet preference (preferred vs. non-preferred).


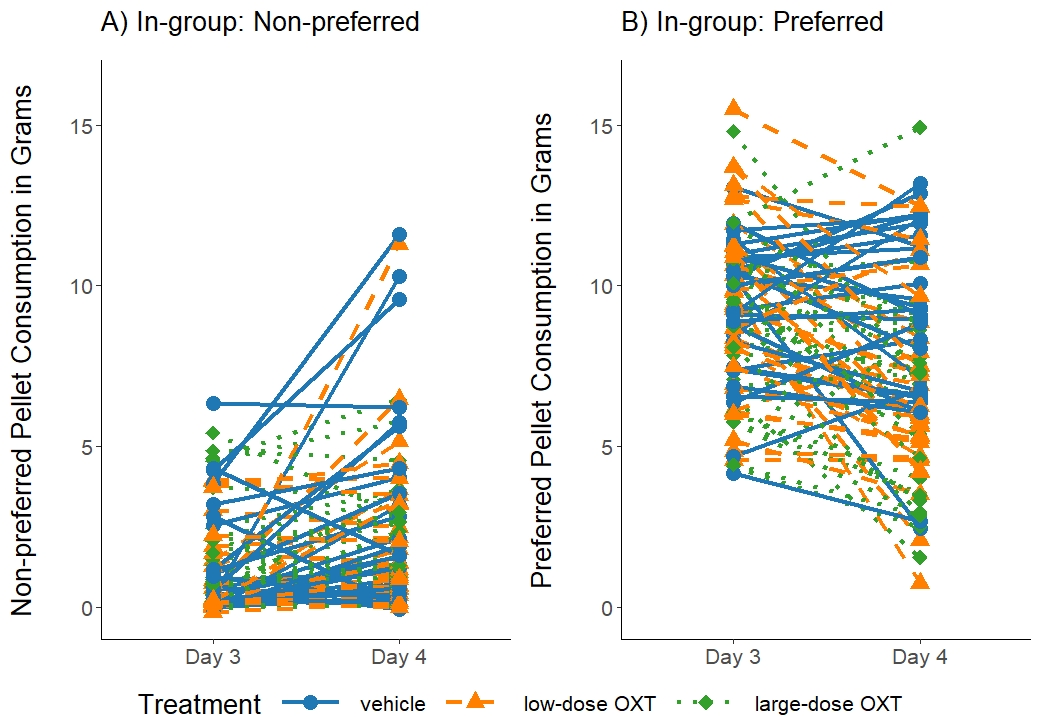


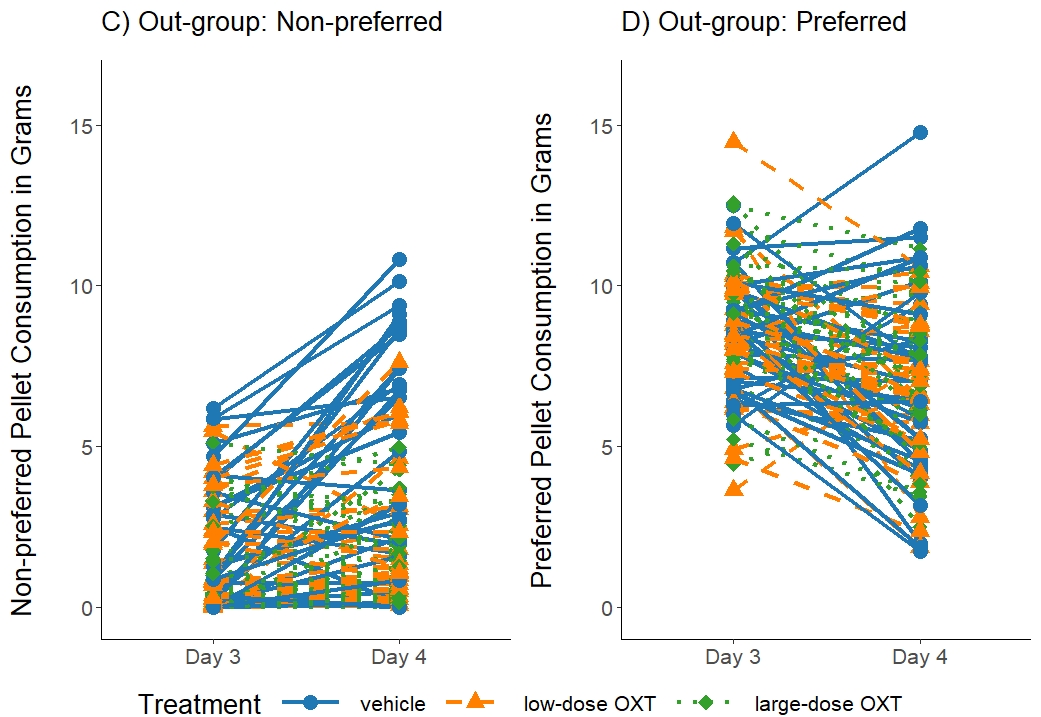


**Fig. 2** Acute OXT effects on STFP. Originally non-preferred pellets consumption (amount consumed in grams) in the in-group (panel A), originally preferred pellets consumption in the in-group (panel B), originally non-preferred pellets consumption in the out-group (panel C), and originally preferred pellets consumption in the out-group (panel D) are displayed. In all panels, the pellet consumption of the vehicle group is represented by the solid blue line and circle symbols, the low-dose OXT group by the dashed orange line and triangles, and the large-dose OXT group by the dotted green line and squared symbols.

**Long-term OXT effects on STFP: Individual data plots**

Here, we display the individual choice behavior of the rats on days 3 (pre-social interaction), day 4 (first post-social interaction testing day), and day 5 (second post-social interaction testing day) for each familiarity group (in- vs. out-group), treatment condition (vehicle vs. low-dose OXT vs. large-dose OXT), and pellet preference (preferred vs. non-preferred).


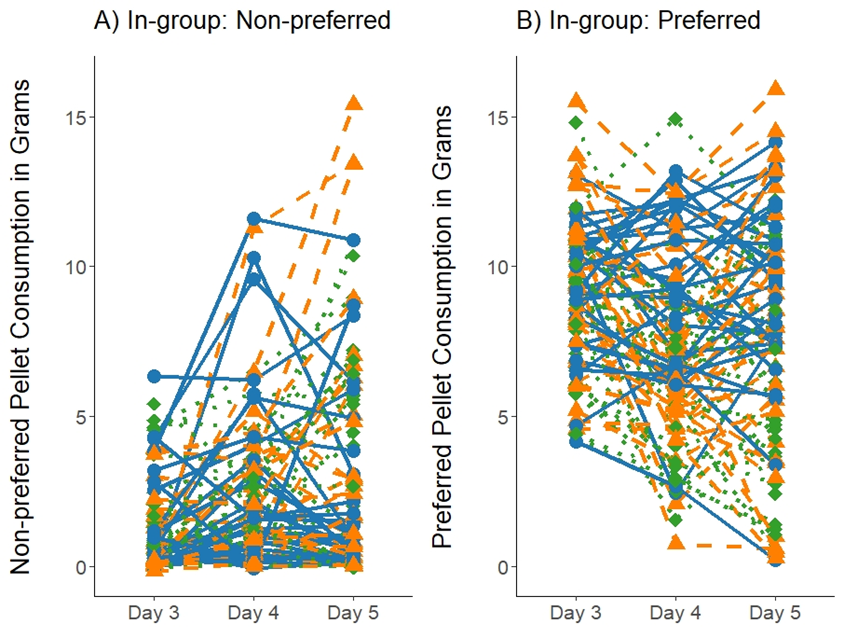


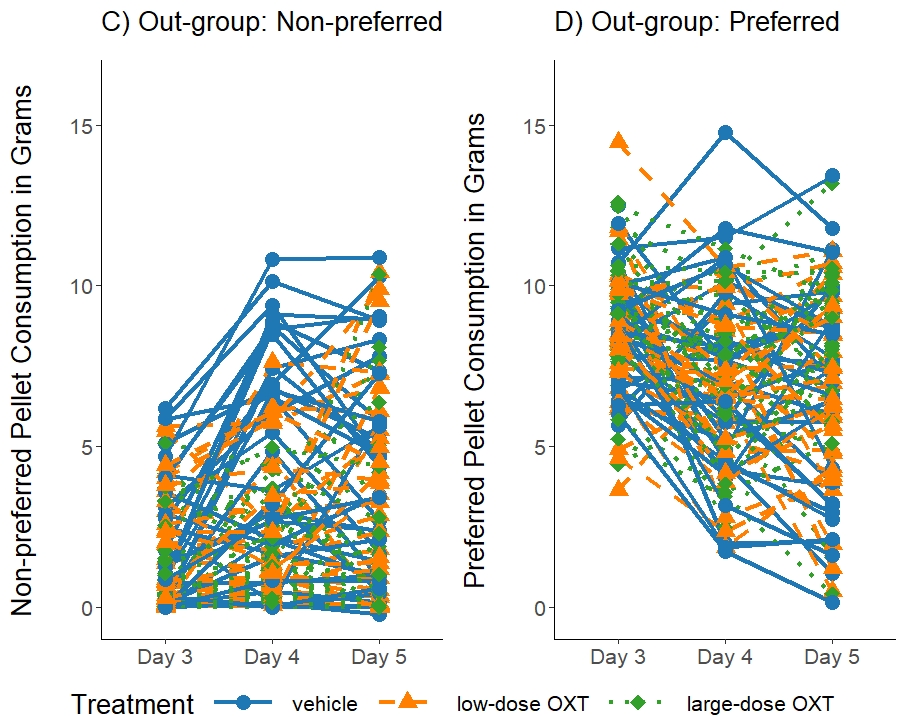


**Fig. 3** Acute and long-term OXT and effects on STFP. Originally non-preferred pellets consumption in the in-group (panel A), originally preferred pellets consumption in the in-group (panel B), originally non-preferred pellets consumption in the out-group (panel C), and originally preferred pellets consumption in the out-group (panel D) are displayed. In all panels, the pellet consumption of the vehicle group is represented by the solid blue line and circle symbols, the low-dose OXT group by the dashed orange line and triangles, and the large-dose OXT group by the dotted green line and squared symbols.

**Oxytocin anorexic effects**

To evaluate whether OXT’s anorexic effects explained our results, we computed the effects of familiarity (in- vs. out-group), treatment (vehicle vs. low-dose OXT vs. large-dose OXT), and day (days 3 vs. 4 vs. 5) on total pellet consumption (originally preferred + non-preferred pellets consumption per day). The analysis showed a significant simple main effect of treatment (*F*_[2, 202]_ = 9.079, *p* = .000) and a significant simple main effect of day (*F*_[2, 404]_ = 16.129, *p* = .000), and a significant interaction effect between treatment and day (*F*_[4, 404]_ = 21.241, *p* = .000). Post-hoc tests indicated that all groups treated with OXT, regardless of the dose, decreased their total pellet consumption on day 4 compared to day 3 (in- low-dose OXT: *t*_[31]_ = 3.69, *p* = .002; in- large-dose OXT: *t*_[35]_ = 5.34, *p* = .000; out- low-dose OXT: *t*_[33]_ = 2.87, *p* = .014; out- large-dose OXT: *t*_[36]_ = 4.86, *p* = .000) and recovered previous levels of consumption on day 5 (day 3 vs. day 5; in- low-dose OXT: *t*_[31]_ = -1.6, *p* = .165; in- large-dose OXT: *t*_[35]_ = 0.276, *p* = .802; out- low-dose OXT: *t*_[33]_ = 0.252, *p* = .802; out- large-dose OXT: *t*_[36]_ = 1.25, *p* = .284). The vehicle groups increased their total pellet consumption from day 3 to day 4 (in- vehicle: *t*_[30]_ = -4, *p* = .001; out- vehicle: *t*_[37]_ = -3.04, *p* = .009) and kept it stable on day 5 (in- vehicle: *t*_[30]_ = 1.1, *p* = .337; out- vehicle: *t*_[37]_ = 2.1, *p* = .064). In line with previous literature, acute OXT decreased palatable total pellet consumption on the same day when vehicle-treated groups increased it. These effects were no longer observable on day 5 (in-group: vehicle vs. low-dose OXT: *t*_[61.5]_ = 0.391, *p* = .836; vehicle vs. large-dose OXT: *t*_[60.9]_ = 2.46, *p* = .061; low-dose OXT vs. large-dose OXT: *t*_[59.9]_ = 1.85, *p* = .207; out-group: vehicle vs. low-dose OXT: *t*_[71.9]_ = 0.848, *p* = .798; vehicle vs. large-dose OXT: *t*_[69.1]_ = 0.97, *p* = .754; low-dose OXT vs. large-dose OXT: *t*_[62.1]_ = 0.014, *p* = .989) unveiling OXT's influence on socially acquired preferences beyond its acute anorexic effects. This analysis indicates that OXT had acute but not long-lasting anorexic effects and that its anorexic effects were independent of the familiarity with the demonstrator.


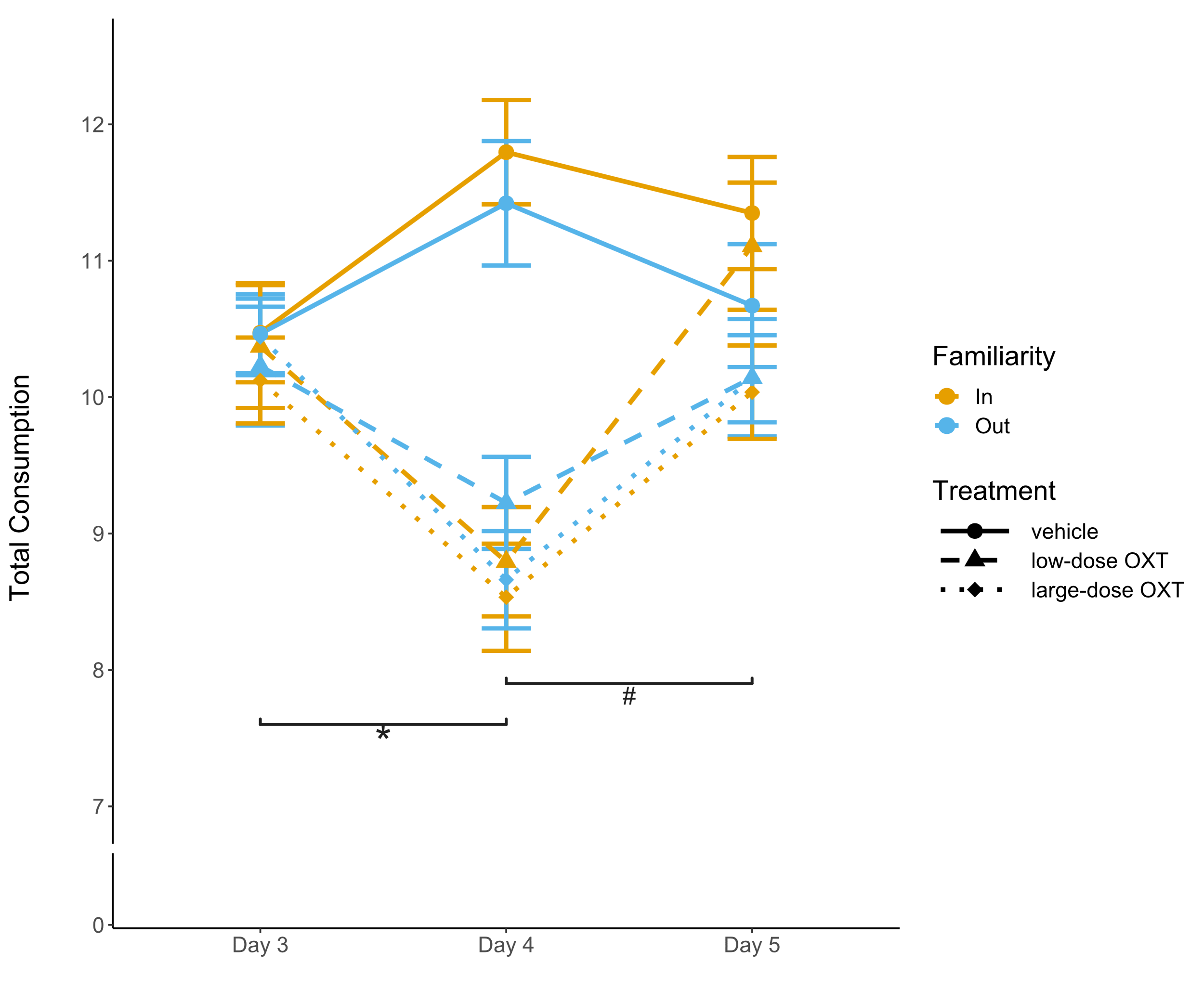


**Fig. 4** OXT effects on total pellet consumption. In-group is represented by orange and out-group by blue. Vehicle groups are represented by the solid line and circle symbols, low-dose OXT groups by the dashed line and triangles, and large-dose OXT groups by the dotted line and squared symbols. The mean (±SEM) of the total amount of pellets consumed (originally preferred + non-preferred pellets) decreased on day 4, immediately after OXT administration, but recovered after OXT dissipated on day 5. Vehicle groups increased their total pellet consumption after social interaction. However, the complex pattern of OXT effects on STFP shown in figures 4 and 5 in the manuscript cannot be solely explained by OXT anorexic effects (see main text for details). * All treatments p < .05; # OXT-treated groups p < .05

To confirm the lack of relationship between the anorexic effects of OXT and the STFP-related shifts in pellet consumption, we ran correlations between the difference in consumption of the originally non-preferred pellets between days 3 and 4 (as a metric for STFP) and the total pellet consumption on that day (anorexic effect) for each familiarity and treatment group (except the vehicle group due to the absence of OXT-related anorexic effects). We also ran correlations between the difference in originally non-preferred pellets consumption between days 4 and 5 and total pellet consumption on day 4. Table 1 shows these correlations (statistically significant correlations are marked in bold). The anorexic effects caused by the large dose of OXT were correlated with the difference in originally non-preferred pellets consumed on days 4 and 5 for both in- and out-groups. We believe that these correlation results support our conclusion that the modulatory effects of OXT and familiarity on STFP cannot be fully explained by an OXT’s anorexic effects.

|  | Orig. non-preferred D4 – D3 with Total D4 | | | | Orig. non-preferred D5 – D4 with Total D4 | | | |
| --- | --- | --- | --- | --- | --- | --- | --- | --- |
|  | In | | Out | | In | | Out | |
|  | Low-dose OXT | Large-dose OXT | Low-dose OXT | Large-dose OXT | Low-dose OXT | Large-dose OXT | Low-dose OXT | Large-dose OXT |
| r | -0.1411 | -0.2588 | -0.0873 | -0.1518 | 0.0265 | **0.2335** | 0.0516 | **0.2513** |
| p-value | 0.2563 | 0.7958 | 0.4676 | 0.1865 | 0.8282 | **0.0452** | 0.6673 | **0.0289** |

**Table 1** The first half (blue column) shows the correlation between the difference in consumption of the originally non-preferred pellets between days 3 and 4 (as a metric for STFP) and the total pellet consumption on that day (anorexic effect). The second (orange column) shows the correlation between the difference in originally non-preferred pellets consumption between days 4 and 5 and total pellet consumption on day 4. The correlations are split by familiarity and treatment group, except the vehicle group (no OXT-related anorexic effects). Correlations (r) and p-values are reported and the statistical significance is highlighted in bold.

**Social interaction: time spent by the observer exploring the demonstrator**

We measured how much time the observers spent socially exploring the demonstrators as a potential mediator for the familiarity and OXT effects on STFP. As shown, the large-dose OXT group explored the demonstrator less than the others (vehicle vs. low-dose OXT: *t*_[149]_ = 0.763, *p* = .447; vehicle vs. large-dose OXT: *t*_[145]_ = 3.55, *p* = .000; low-dose OXT vs. large-dose OXT: *t*_[148]_ = 2.7, *p* = .012). However, familiarity did not modulate social interaction time (*F*_[1, 224]_ = 1.364, *p* = .244). We therefore conclude that the OXT-related differences in social exploration time cannot explain the complex pattern of results shown in the STFP task.

**
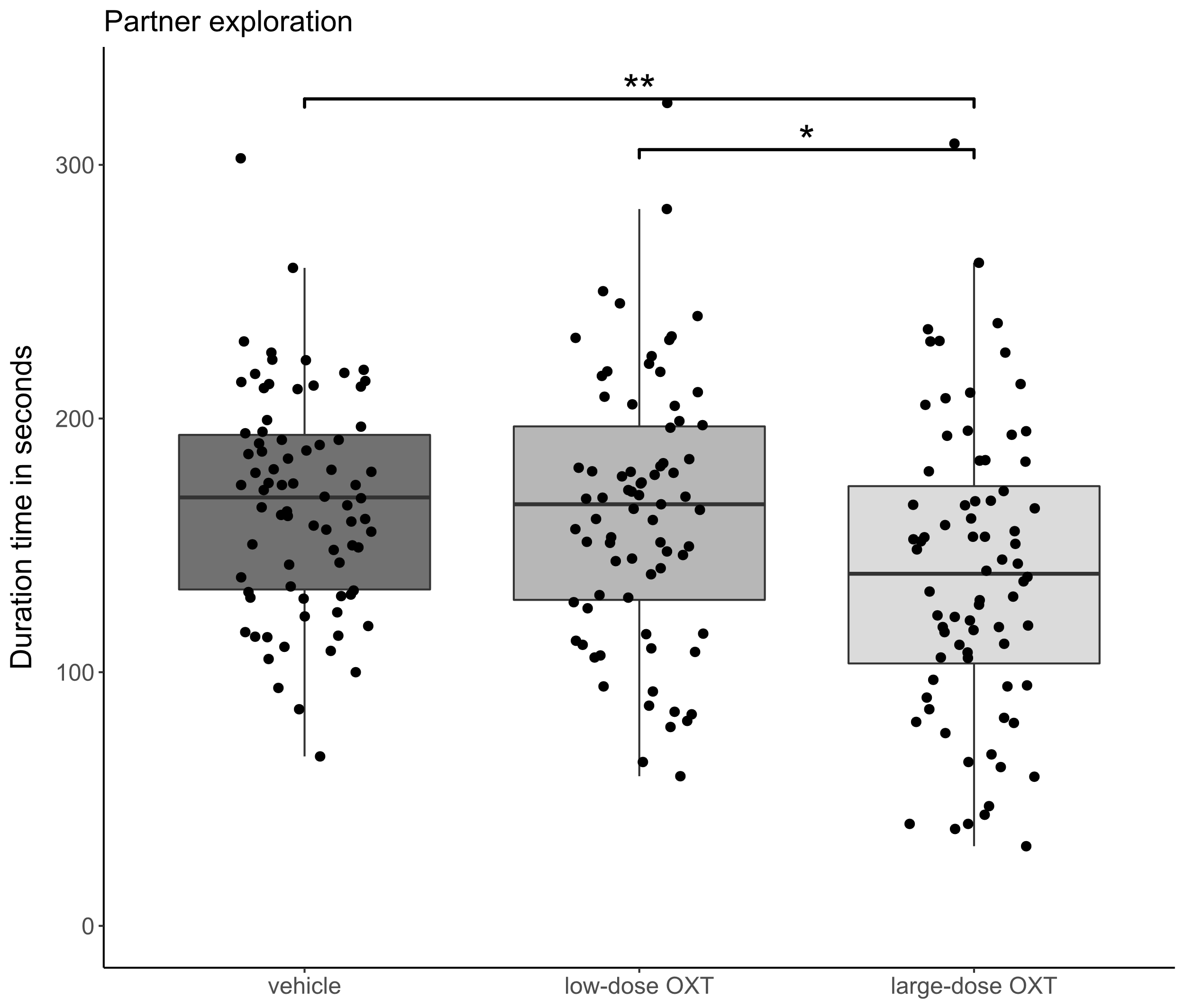
**

**Fig. 5** OXT effects on observers’ exploration time. The horizontal line inside the boxes represents the median time spent by the observers exploring the demonstrators during social interaction, the hinges represent the 25^th^ and 75^th^ percentiles, and the whiskers represent the interquartile ranges. Dots represent individual data points. The observers’ exploration time of the large-dose OXT group was shorter than the other treatment groups. * p < .05; ** p < .01
